# Enhancing Early Diagnosis: Multimodal AI Approaches for Neurodegenerative Diseases

**DOI:** 10.26502/jbb.2642-91280211

**Published:** 2026-03-10

**Authors:** Aneesh Swamy, Devendra K Agrawal

**Affiliations:** Department of Translational Research, College of Osteopathic Medicine of the Pacific, Western University of Health Sciences, Pomona, California 91766 USA

**Keywords:** Artificial intelligence (AI), AI-Driven Biomarkers, Digital Phenotyping, Early detection, Electrophysiology (EEG), Explainable AI (XAI), Longitudinal Monitoring, Multimodal Fusion, Neurodegenerative Diseases, Neuroimaging (MRI/PET), Predictive Modeling

## Abstract

Neurodegenerative diseases such as Alzheimer’s and Parkinson’s impose a staggering global burden, yet timely identification remains hindered by a fundamental mismatch between the slow unfolding of pathology and the static nature of traditional diagnostic frameworks. While conventional clinical markers often fail to identify decline until irreversible neuronal loss has occurred, artificial intelligence (AI)-driven biomarkers derived from neuroimaging, electrophysiology, and digital phenotyping offer a transformative proactive paradigm. This review evaluates how machine-learning models extract high- dimensional, subvisual patterns from MRI, PET, and EEG datasets to detect preclinical deviations that outpace traditional markers in predictive timelines. We argue that the primary value of these technologies lies in a categorical shift toward continuous, temporally informed disease modeling designed to fill the “detection gap” between early protein accumulation and overt clinical impairment. By synthesizing evidence across various modalities, we highlight the superior performance of multimodal fusion architectures in capturing the biological complexity of neurodegeneration. However, clinical translation faces significant hurdles, including data heterogeneity, the “black-box” nature of deep learning, and the necessity for global equity in dataset representation. Ultimately, by integrating explainable AI with longitudinal data streams, these biomarkers can redefine neurodegenerative care-transforming diagnosis from a reactive confirmation of damage into a precise tool for risk stratification, trial enrichment, and early therapeutic intervention.

## Introduction

Neurodegenerative diseases such as Alzheimer’s disease, Parkinson’s disease, and amyotrophic lateral sclerosis, as well as related syndromes such as frontotemporal dementia [1,2] continue to impose a substantial global burden, affecting cognitive, functional, and social well-being while contributing significantly to morbidity and healthcare costs. Early detection remains critical because disease- modifying therapies are most effective when initiated during preclinical or early symptomatic stages, before irreversible neuronal loss has occurred [3–5]. Traditional clinical markers, however, fall short in providing timely identification. Symptom-based diagnosis, including motor impairment or measurable cognitive decline, tends to (including in mild cognitive impairment and other prodromal syndromes) [6,7] emerge only after degeneration has progressed extensively [8,9]. Even established biomarkers such as tau or amyloid-ß (Aß) ratios in the cerebrospinal fluid (CSF), and genetic testing for known pathogenic variants, offer improved diagnostic precision but remain invasive, costly, or insufficiently sensitive in the earliest disease phases [9–13].

These limitations reveal a fundamental mismatch between the temporal dynamics of neurodegenerative pathology and the tools currently used to detect it. Neurodegeneration unfolds over years to decades, yet most diagnostic frameworks remain anchored to static clinical snapshots taken late in the disease course. Closing this temporal gap requires biomarkers capable of detecting subtle, distributed, and dynamic biological changes well before overt symptom emergence.

In contrast, advances in artificial intelligence have enabled the detection of subtle neurobiological deviations that may precede symptomatic presentation by years. Machine-learning models applied to MRI, PET, and EEG datasets are uncovering preclinical patterns that are imperceptible to the human eye [8,14,15]. Despite these advances, very few studies have directly evaluated whether artificial intelligence (AI)-derived imaging or electrophysiological signatures can consistently outpace traditional biomarkers in predictive timelines, leaving a significant gap in understanding how these emerging technologies should be positioned clinically [14,15].

Beyond isolated performance gains, a central unanswered question remains whether AI-driven biomarkers represent incremental improvements within existing diagnostic paradigms or a categorical shift toward temporally informed, continuous disease modeling. Resolving this distinction is critical, as it determines whether AI tools should function as adjuncts to conventional diagnostics or as foundational components of early detection, risk stratification, and trial enrichment.

**Figure 1:**
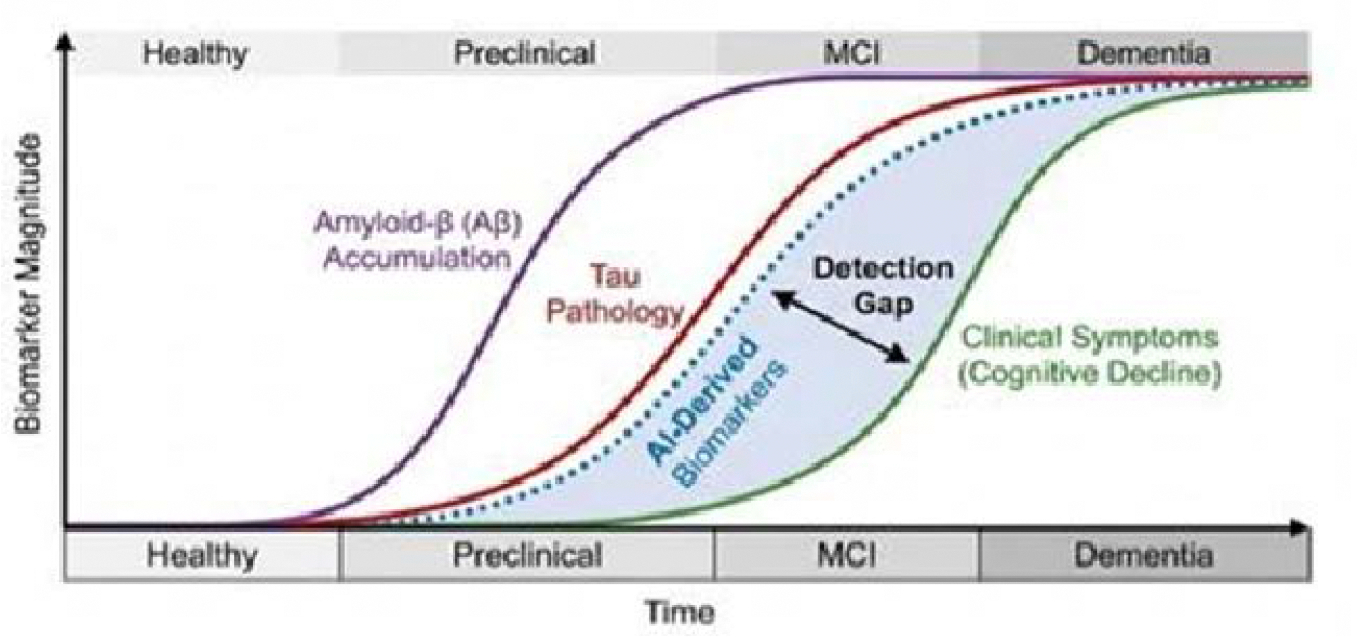
Hypothetical model of dynamic biomarkers of the Alzheimer’s pathological cascade. AI and digital biomarkers (indicated) aim to fill the detection gap between early protein accumulation (Aβ, Tau) and the onset of clinical impairment.

In this critical review article, we present rationale for the thesis that AI-derived biomarkers from neuroimaging, electrophysiology, and digital phenotyping collectively redefine how early neurodegenerative disease can be detected, monitored, and operationalized in clinical and research settings. Rather than focusing solely on classification accuracy, we emphasize temporal lead-time, biological interpretability, and clinical actionability as the defining dimensions by which these biomarkers should be evaluated. By synthesizing evidence across imaging-based, EEG-based, and multimodal AI approaches, this work aims to clarify where AI-driven biomarkers meaningfully outperform traditional markers, where they remain limited, and how they can be responsibly integrated into future diagnostic frameworks (Figure 1).

### Current Imaging-Based AI Biomarkers

AI-enhanced neuroimaging represents one of the most mature and clinically advanced domains of AI-driven biomarker development for neurodegenerative disease [16–18]. Unlike traditional radiologic assessment, which relies on visually appreciable structural abnormalities, AI-based imaging biomarkers are designed to detect high-dimensional spatial patterns and subtle deviations distributed across the brain. This capability allows imaging data to be leveraged not merely for diagnosis, but for modeling disease trajectory and estimating risk before overt clinical decline.

Structural MRI analyses employing convolutional neural networks now quantify gray matter atrophy, cortical thinning, and hippocampal volume loss with increasing precision, allowing for earlier differentiation between healthy aging and pathological decline [19]. Rather than focusing on single-region volumetrics, contemporary models learn distributed atrophy signatures that reflect network-level vulnerability, improving sensitivity to early disease states.

Diffusion Tensor Imaging extends this capability by identifying microstructural white-matter degeneration through alterations in fractional anisotropy and mean diffusivity, parameters that AI models can map longitudinally to predict disease conversion [20]. These microstructural changes often precede macroscopic atrophy, positioning diffusion-based AI biomarkers as potential indicators of early axonal and connectivity disruption.

Functional MRI provides additional insight by capturing disruptions in intrinsic connectivity networks; graph-based machine-learning algorithms detect aberrant network topology and connectivity patterns that may precede overt cognitive changes [14,15]. Such models shift the focus from localized damage to systems-level dysfunction, aligning more closely with contemporary views of neurodegeneration as a network disease rather than a focal process.

PET imaging further complements structural and functional techniques. AI models analyzing FDG- PET reveal early hypometabolic patterns, while amyloid and tau PET studies highlight preclinical pathological accumulation that machine-learning systems can quantify with high sensitivity [21–24]. Importantly, AI-based PET analysis enables continuous risk estimation across pathological burden, rather than reliance on binary positivity thresholds. Increasingly, multimodal fusion approaches combine MRI, PET, clinical variables, and demographic factors to generate comprehensive predictive frameworks that outperform single-modality analyses and offer a more nuanced view of disease risk [15,25].

These approaches reflect a growing recognition that no single imaging modality captures the full biological complexity of neurodegeneration, and that integrated representations are necessary to achieve robust early prediction. Collectively, imaging-based AI biomarkers derive their strength not only from improved classification accuracy, but from their ability to extract temporally informative signals embedded within high-dimensional neuroimaging data. By identifying distributed and subvisual changes that precede clinical symptoms, these models establish neuroimaging as a cornerstone of AI-driven early detection strategies, while also highlighting the importance of multimodal integration to mitigate modality- specific limitations.

### EEG-Based AI Biomarkers

While imaging-based AI biomarkers excel at capturing spatially distributed structural and metabolic changes, electrophysiological biomarkers provide a fundamentally different and complementary window into neurodegeneration by directly measuring neural dynamics in real time. EEG-based AI models therefore occupy a distinct position within the biomarker landscape, prioritizing temporal sensitivity and functional disruption rather than anatomical localization.

While imaging provides spatial resolution, electrophysiological and digital tools offer superior temporal precision, capturing dynamic functional changes. EEG-based deep learning models are now capable of distinguishing neurodegenerative profiles from healthy controls by analyzing spectral power density and coherence abnormalities. Rather than reflecting accumulated tissue damage, these electrophysiological signatures often index early circuit-level dysfunction, which may emerge before detectable structural or metabolic abnormalities.

In Parkinson’s disease, for instance, AI algorithms have successfully isolated the dominance of beta- frequency power in the subthalamic nucleus, a neural signature characteristic of bradykinesia [26–30], even in the absence of overt tremor. This highlights a key advantage of EEG-derived biomarkers: their ability to detect disease-relevant physiological states that fluctuate dynamically and may not be captured by static imaging modalities.

Beyond controlled clinical environments, EEG-based AI biomarkers align naturally with the growing emphasis on scalable, longitudinal monitoring. Their noninvasive nature and relative affordability make them particularly well suited for repeated assessments, enabling high-frequency sampling of disease- related neural activity over time [31]. Home-based, unobtrusive monitoring systems and web-enabled conversational interactions have also been explored as scalable approaches to capture continuous functional change and relate digital measures to underlying neuropathology [32,33]. This temporal granularity positions EEG as a potential early indicator of disease progression, treatment response, or imminent clinical transition.

**Figure 2:**
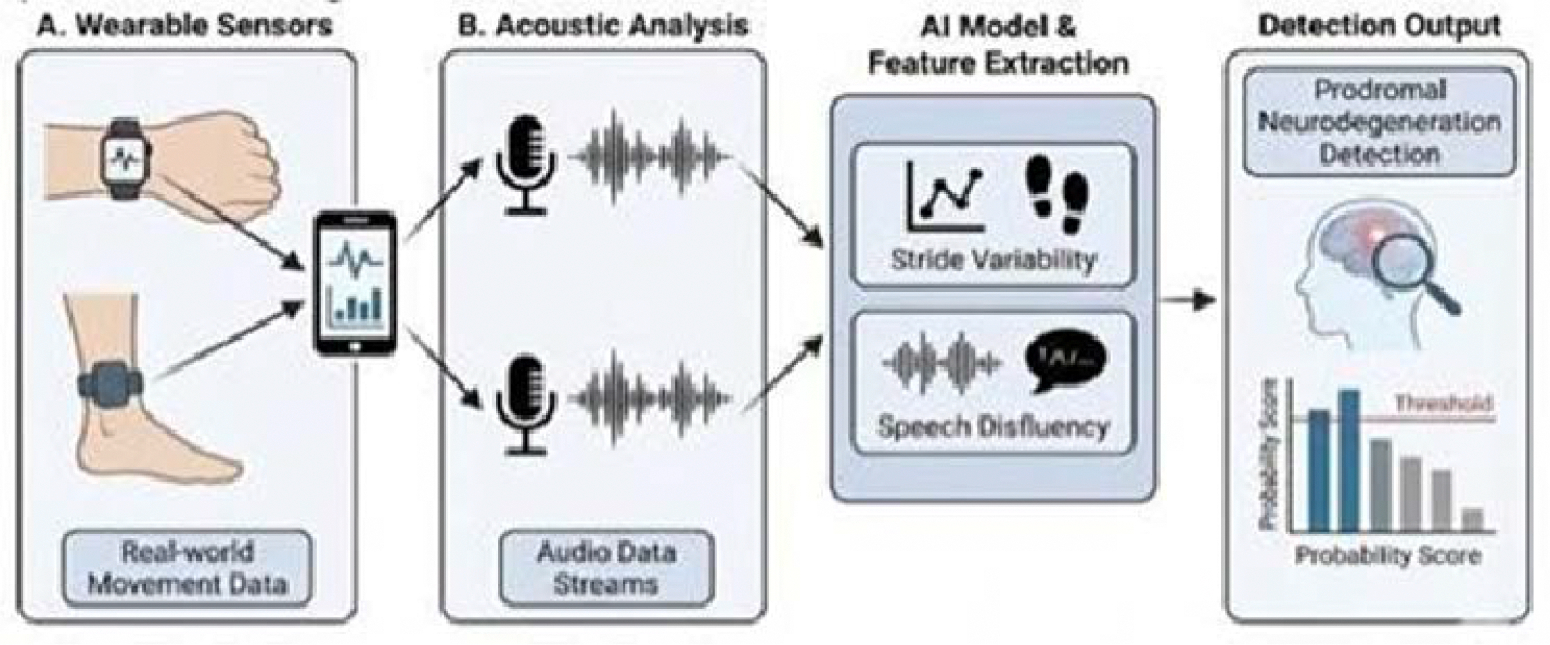
Wearable sensors (A) and acoustic analysis (B) provide high frequency, real-world data streams. AI models extract features such as stride variability and speech disfluency to detect prodromal neurodegeneration.

Beyond the clinic, the rise of digital biomarkers-data collected from wearable devices and smartphones-promises scalable, cost-effective screening [34–39]. Machine-learning analysis of data from smartphone accelerometers and gyroscopes can detect minute gait irregularities and reduction in arm swing years before a clinical diagnosis is possible [40–48]. Similarly, acoustic analysis of speech signals has emerged as a potent tool for Alzheimer’s screening, with natural language processing (NLP) models identifying prosodic pauses and linguistic complexity reductions that correlate strongly with CSF amyloid levels [35,36,38,39,49] (Figure 2).

Importantly, EEG and digital biomarkers should not be viewed as replacements for imaging-based AI markers, but rather as complementary signals that capture different biological dimensions of neurodegeneration. Whereas imaging reflects cumulative structural and pathological burden, electrophysiological and behavioral markers are sensitive to real-time network instability and functional compensation. Integrating these modalities therefore enables a more complete representation of disease trajectory, combining spatial specificity with temporal responsiveness.

### Comparison with Traditional Clinical Markers

When compared with conventional diagnostic approaches, AI-driven imaging and EEG biomarkers offer a fundamentally different mode of disease detection, shifting emphasis from late-stage confirmation to early trajectory identification. Traditional clinical markers are largely designed to identify established disease states, whereas AI-derived biomarkers aim to detect subtle deviations that signal impending pathological transition.

Cognitive assessments such as the Mini-Mental State Examination (MMSE) and Montreal Cognitive Assessment test (MoCA) remain important for staging but lack sufficient sensitivity for prodromal or minimally symptomatic phases; studies indicate the MMSE detects as few as 18% of Mild Cognitive Impairment cases [50], often identifying decline only after significant neuronal loss has occurred. These tools are inherently constrained by their reliance on overt behavioral expression, which reflects downstream consequences of neurodegeneration rather than its earliest biological drivers.

Biochemical assays including CSF tau and Aß42 measures, although clinically validated, require invasive lumbar punctures and may reflect downstream consequences rather than the earliest pathological events [9,51]. Recent advances in plasma and CSF assays, including plasma Aβ and phosphorylated tau species, demonstrate strong diagnostic performance and improved prediction of longitudinal decline, while consensus and guideline efforts are accelerating standardization for clinical use [52–58]. While such markers provide valuable molecular specificity, their clinical utility is limited by accessibility, patient burden, and restricted feasibility for repeated longitudinal monitoring.

Similarly, standard functional assessments of gait or speech typically demonstrate observable abnormalities only after 50–70% of nigral dopaminergic neurons have effectively been compromised [59]. By the time these deficits become clinically apparent, opportunities for disease-modifying intervention may already be substantially diminished.

In this context, AI-driven biomarkers provide a unique advantage by detecting structural, metabolic, and electrophysiological disruptions prior to clinically apparent impairment. Crucially, these systems operate on continuous biological signals rather than threshold-based clinical cutoffs, enabling earlier risk stratification and finer-grained tracking of disease evolution.

Rather than replacing traditional markers outright, AI-based biomarkers are best understood as redefining their role within the diagnostic pipeline. Clinical assessments and biochemical tests retain value for confirmation, staging, and phenotypic characterization, while AI-driven imaging and electrophysiology extend detection upstream into preclinical and prodromal phases. This complementary positioning underscores a broader paradigm shift from episodic diagnosis toward continuous, temporally informed disease modeling (Table 1).

### Integration and Predictive Modeling

Efforts to improve early detection now emphasize integrated predictive modeling, in which AI algorithms synthesize imaging, electrophysiology, clinical characteristics, and digital biomarkers to produce unified risk-stratification systems. This shift reflects a growing recognition that neurodegeneration cannot be captured by any single modality, as structural, metabolic, functional, and behavioral changes evolve along partially independent trajectories.

Multimodal integration enhances predictive accuracy by capturing complementary aspects of disease physiology; recent frameworks demonstrating this approach have shown that fusing MRI, PET, and clinical data yields significantly higher classification performance than any single modality [60]. Importantly, the advantage of integration extends beyond performance metrics, enabling models to infer disease state from convergent biological signals rather than relying on isolated abnormalities.

**Figure 3:**
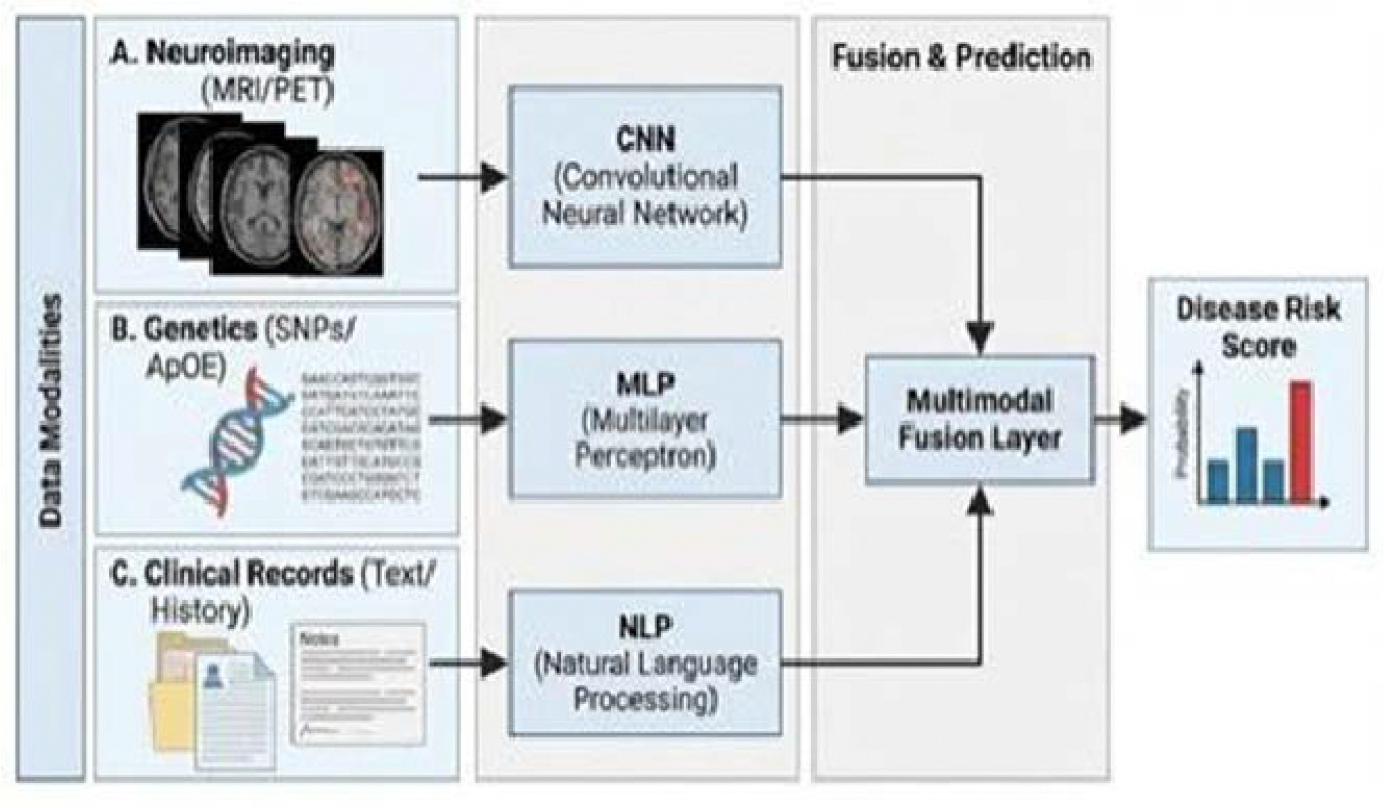
Integrated Predictive Modeling: Multimodal Fusion Architecture.

Cross-modality learning, in which AI systems jointly analyze structural changes, metabolic deficits, and even genetic data, allows for early detection of complex disease trajectories and produces more individualized predictions [15,25]. By learning shared latent representations across modalities, these models are better equipped to account for heterogeneity in disease onset, progression rate, and clinical phenotype (Figure 3).

Transfer-learning approaches have become particularly critical in this domain, enabling researchers to adapt pretrained models to neurodegenerative datasets that are often too small for conventional deep- learning frameworks [61]. This strategy not only improves model stability but also facilitates the reuse of learned biological features across related disease contexts, accelerating translational progress.

Furthermore, unsupervised techniques are beginning to uncover latent disease subtypes and progression patterns not identifiable through clinical evaluation alone [62]. Such approaches challenge traditional diagnostic categories by revealing continuous spectrums of pathology, offering a data-driven basis for refining disease taxonomy and stratifying patients more precisely.

Integrating molecular biomarkers or genetic risk alleles with imaging features has further improved early risk prediction for individuals likely to convert from mild cognitive impairment to dementia [63,64]. Together, these advances position predictive modeling not simply as a tool for early diagnosis, but as a framework for anticipating disease trajectory, informing trial enrollment, and tailoring intervention timing. Rather than treating multimodal integration as an optional enhancement, these findings suggest it should be considered foundational to AI-driven neurodegenerative biomarker development. Only by combining spatially resolved imaging, temporally sensitive electrophysiology, molecular risk indicators, and clinical context can predictive models approximate the complexity of neurodegenerative disease biology (Figure 3).

### Challenges and Limitations

Despite substantial advances, several challenges hinder clinical translation of AI-based neurodegenerative biomarkers. Imaging and EEG datasets are highly variable, with differences in acquisition protocols and scanner characteristics contributing to inconsistent performance. To address this, recent efforts have focused on statistical harmonization techniques such as ComBat to remove site- specific effects while preserving biological variability [65].

While harmonization methods mitigate inter-site variability, they do not fully resolve deeper issues related to dataset composition, population bias, and incomplete representation of disease heterogeneity. Models trained on narrowly defined cohorts may perform well under controlled conditions yet fail to generalize across clinical settings, limiting their reliability in real-world deployment.

Model interpretability remains a central limitation; many deep-learning systems operate as “black boxes,” making it difficult for clinicians to verify that predictions rely on biologically meaningful features. However, emerging “explainable AI” (XAI) frameworks are beginning to bridge this gap. For instance, recent constrained deep-learning models have successfully identified novel, biologically valid biomarkers in Alzheimer’s disease by enforcing interpretability constraints during training [66].

Interpretability is not merely a technical preference but a prerequisite for clinical trust, regulatory acceptance, and ethical deployment. Without transparent reasoning pathways, AI-driven biomarkers risk remaining confined to research settings, unable to inform high-stakes clinical decisions such as early intervention or trial eligibility.

Additional challenges arise from the longitudinal nature of neurodegenerative disease. Many AI models are trained on cross-sectional data, limiting their ability to capture within-patient trajectories or adapt to evolving disease states. This mismatch between static model design and dynamic disease progression constrains the predictive utility of otherwise high-performing systems.

Finally, integration into clinical workflows presents practical and ethical hurdles. AI-based biomarkers must coexist with established diagnostic practices, operate within time and resource constraints, and align with clinician decision-making processes. Addressing these challenges will require not only technical innovation, but also prospective validation, interdisciplinary collaboration, and careful consideration of how AI outputs are communicated and acted upon in real-world care [67,68].

### Future Directions

The future of neurodegenerative disease management lies in the shift from static diagnostic snapshots to continuous, longitudinal monitoring. AI models that integrate real-time digital phenotyping with periodic imaging assessments are poised to track disease progression with granular precision, potentially serving as sensitive surrogate endpoints for clinical trials [69,70]. By prioritizing longitudinal data streams, these approaches directly address the limitations of cross-sectional modeling and better reflect the progressive nature of neurodegenerative pathology.

To overcome challenges related to data heterogeneity and limited generalizability, future efforts must emphasize large-scale, multi-site, and prospectively collected datasets. Federated learning and privacy- preserving model training offer promising pathways for aggregating diverse data without centralized data sharing, enabling models to learn from heterogeneous populations while maintaining data governance standards. Such strategies are critical for ensuring that AI-driven biomarkers remain robust across healthcare systems and demographic contexts.

Interpretability will play a central role in determining whether AI biomarkers transition from research tools to clinically actionable instruments. The integration of explainable AI methods into model design, rather than post hoc interpretation, allows clinicians to assess whether predictions align with known disease mechanisms and individual patient context. Embedding transparency at the model level also facilitates regulatory review and supports clinician confidence in high-stakes decision-making.

This transition is supported by evolving regulatory frameworks; the FDA’s “Software as a Medical Device” (SaMD) action plan specifically outlines pathways for adaptive AI algorithms that update continuously based on real-world data [71]. These frameworks acknowledge the need for iterative learning systems while emphasizing safeguards for performance monitoring, bias detection, and clinical oversight.

Moreover, achieving global equity requires that future algorithms be trained on diverse, globally representative populations to ensure that AI-driven diagnostics do not perpetuate existing healthcare disparities [72]. Addressing bias is not solely a data problem, but a design imperative that requires deliberate inclusion strategies, continuous auditing, and transparent reporting of model limitations.

Ultimately, the convergence of longitudinal modeling, interpretability-driven design, and inclusive data practices positions AI-driven biomarkers to move beyond proof-of-concept demonstrations toward meaningful clinical integration. By aligning technical innovation with regulatory, ethical, and clinical realities, future AI systems can fulfill their promise of enabling earlier detection, personalized risk assessment, and more effective intervention in neurodegenerative disease.

## Conclusions

AI-driven biomarkers derived from neuroimaging and EEG offer strong potential for detecting neurodegenerative diseases earlier and with greater accuracy than traditional clinical or biochemical markers, providing a foundation for a proactive rather than reactive diagnostic paradigm [8,14,15]. Despite this promise, meaningful clinical translation continues to be challenged by limitations in data standardization, external validation, and model interpretability, all of which must be addressed to ensure reliable patient-facing deployment [14,15,25].

Multimodal AI approaches that integrate MRI, PET, EEG, and clinical variables have consistently shown superior performance over single-modality systems, underscoring the importance of comprehensive cross-domain fusion for early detection [8,14,15]. Deep-learning models are increasingly sensitive to subtle structural and network-level changes that appear long before abnormalities are evident on cognitive tests or CSF assays, supporting their usefulness in identifying preclinical disease trajectories [14,15]. These systems also demonstrate measurable advantages in predictive timelines, often detecting pathological signals years before conventional diagnostic markers reach abnormal thresholds [8,15]. Furthermore, the scalability and noninvasiveness of EEG position AI-enhanced electrophysiologic biomarkers as promising tools for population-level screening and longitudinal monitoring [8,14].

Taken together, these findings suggest that the primary value of AI-driven biomarkers lies not merely in improved classification performance, but in their ability to reorient neurodegenerative disease detection around time, trajectory, and individualized risk. The transition from episodic diagnosis to continuous disease modeling has profound implications for clinical trial design, preventive interventions, and health system planning.

As model accuracy improves, these biomarkers hold potential for individualized risk stratification and identifying candidates for neuroprotective clinical trials [8,15]. Ultimately, achieving clinical adoption will require large, diverse datasets and advancing transparency within AI systems to ensure clinician trust and ethical integration into routine care. The success of AI-driven biomarkers should therefore be judged not only by technical performance, but by their capacity to meaningfully alter disease timelines and enable earlier, more effective intervention.

## Figures and Tables

**Table 1:** Summary of Representative AI-Driven Biomarker Evidence in Neurodegenerative Disease.

Modality	Dataset Context	Performance Summary	Temporal Advantage	Key Limitation
Structural MRI + CNN	Multi-cohort MRI	High AD vs control classification using distributed atrophy patterns	Detects network-level atrophy before overt cognitive decline	Scanner/site variability
Diffusion Tensor Imaging	DTI (FA, MD metrics)	Accurate classification via microstructural white-matter features	Microstructural changes precede macroscopic atrophy	Small datasets
FDG-PET + Deep Learning	18F-FDG PET	High diagnostic accuracy via hypometabolic pattern detection	Identifies metabolic dysfunction before major structural loss	Cost, accessibility
Multimodal Fusion (MRI + PET + Clinical)	Integrated multi-modal datasets	Consistently outperforms single modalities	Enhances early trajectory prediction via convergent signals	Data heterogeneity
Transfer Learning	Pretrained CNN adaptation	Improves performance in limited datasets	Enables early modeling despite small sample sizes	Domain shift bias
Unsupervised Subtyping	Longitudinal cohorts	Identifies latent subtypes and staging patterns	Detects continuous disease spectrum pre-classification	Requires longitudinal data
EEG Spectral Analysis	Beta oscillation analysis	Identifies beta dominance linked to bradykinesia	Captures real-time circuit dysfunction before tremor	Limited screening scalability
Smartphone Motor Biomarkers	Accelerometer/gyroscope data	Detects subtle gait abnormalities	Identifies motor deviation before formal diagnosis	Device variability
Speech / NLP Biomarkers	Narrative speech datasets	Linguistic features distinguish AD from controls	Early speech alterations correlate with pathology	Language variability
Cognitive Screening (MMSE)	Clinical testing	Detects ~18% of MCI cases	Detects impairment after substantial neuronal loss	Low prodromal sensitivity
Molecular Biomarkers (CSF Aβ, Tau)	CSF / blood assays	High specificity for established pathology	Reflect downstream pathology	Invasive; limited scalability
Imaging Harmonization	Cross-scanner datasets	Reduces site variability while preserving signal	Improves model reliability across sites	Does not resolve population bias

## Key Points:

- The Clinical Detection Gap: Traditional symptom-based diagnoses and clinical markers often emerge only after extensive, irreversible neuronal loss has occurred, creating a critical mismatch with the decade-long progression of neurodegenerative pathology.
- AI’s Predictive Advantage: Machine-learning models can detect subtle, high-dimensional neurobiological deviations in imaging and electrophysiology that may precede clinical symptoms by several years.
- Enhanced Neuroimaging: Beyond simple volumetrics, AI quantifies distributed atrophy signatures and microstructural white-matter changes that serve as early indicators of axonal and connectivity disruption.
- Temporal Sensitivity of EEG: While imaging offers spatial resolution, EEG-based AI provides real- time functional insights, capturing circuit-level dysfunctions that often emerge before structural damage becomes visible.
- Digital Phenotyping and Scalability: Wearable sensors and acoustic analysis of speech enable low-cost, high-frequency monitoring of “real-world” data, such as gait irregularities and linguistic changes.
- Superiority of Multimodal Fusion: Integrating diverse data streams-including MRI, PET, genetics, and clinical records-consistently outperforms single-modality systems and better accounts for patient heterogeneity.
- Diagnostic Sensitivity Comparison: Traditional tools like the MMSE lack the sensitivity required for early detection, with studies showing they may identify as few as 18% of Mild Cognitive Impairment cases.
- The Necessity of Interpretability: To move beyond “black box” systems and gain clinical trust, the field is transitioning toward Explainable AI (XAI) frameworks that rely on biologically meaningful features.
- Data Harmonization and Equity: Overcoming site-specific variability through statistical harmonization and ensuring globally representative datasets are vital for the responsible, equitable deployment of AI tools.
- Future Paradigm Shift: The field is moving from episodic diagnostic snapshots toward a framework of continuous longitudinal monitoring, which could eventually serve as surrogate endpoints for clinical trials.
